# Hospital and Institutional News

**Published:** 1919-05-24

**Authors:** 


### HOSPITAL AND INSTITUTIONAL NEWS.

### EDITH CAVELL'S LIFE HISTORY.

The present issue of The Hospital includes on pages 177-188 a Special Supplement containing a full study of Edith Cavell's Life History and its Lessons, her Home-coming and Burial, with many illustrations. We felt that our readers would value the means of permanently preserving in a complete form the history of this noble woman, with the scenes of paramount interest attaching to her home-coming and burial last week. All who wish to possess a copy of this Special Supplement should write, without delay, to the Manager of The Hospital, 28-29 Southampton Street, Strand, London, W.C.

### THE PRINCE AS HOSPITAL PRESIDENT.

The election of the Prince of Wales to the post of President of Guy's Hospital is not only according to precedent, but a new link in a great tradition. While the expansion of the voluntary system may be said to date from the time when King Edward and Queen Alexandra, before they ascended the throne, first showed an active interest in it, Guy's Hospital has been closely associated with the Royal Family. In becoming the hospital's President the Prince of Wales succeeds King Edward and King George, and since His Majesty's coronation the post has been provisionally held by Mr. H. Cosmo O. Bonsor until the appropriate date arrived to make the present election. One of the marks of the voluntary system is the individual character and tradition which each hospital contributes to the tradition of the whole. If the Royal Family is associated with several institutions, the Heir Apparent is particularly associated with Guy's. This fact gives to it a precedence worthy of its reputation, which in no way conflicts with the claims of other hospitals by right of age, foundation, or medical prestige. At a time when the voluntary principle in most departments of life is falling into temporary disfavour with the crowd, we may take a proud pleasure in recording that inequality between equals which distinguishes the voluntary hospitals to the lasting good of patients and staff.

### ROYAL ARMY MEDICAL CORPS.

At St. Paul's Cathedral on June 12 at noon will be held a Memorial Service in memory of those officers and men of the R.A.M.C. who fell in the war. We are informed that all applications for tickets should be sent to Capt. A. R. Wright, D.S.O., R.A.M.C., Personal Assistant to Director-General, Army Medical Services, War Office, Adastral House, Victoria Embankment, and should reach him before June 1.

### RED CROSS LEAGUE.

We note that it has been decided to offer the post of Director-General of the newly formed League of Red Cross Societies to a British subject, and that Lieut.-General Sir David Henderson, K.C.V.O., K.C.B., D.S.O., has accepted the position. The magnificent work already accomplished by all Red Cross organisations of the Allies is appreciated in every land, and we should realise the honour which by this appointment is paid to Great Britain.

### THE NAVAL SERVICE.

It is understood that at the end of this month Surgeon Vice-Admiral Sir W. H. Norman, K.C.B., will at his own request vacate the office of Director-General of the Medical Department of the Royal Navy. He has held this post for the last two years, and as those who have acted under him well know, his administration has deserved the greatest praise. It is stated his successor will be Surgeon Captain Sir Robert Hill, K.C.M.G., C.B., who was well known as the principal medical officer of the Grand Fleet in the war period.

### THE DISTRESSED SAILOR.

It is now about two years since King George's Fund for Sailors was started, since when over £110,000 has been distributed among marine benevolent institutions. Every institution or fund for the benefit of the sailor and the seamen is, *primâ facie*, eligible for a grant, and King George's Fund is already proving a magnet for the individual marine charities, as King Edward's Fund has proved for the many hospitals of London. Avowedly modelled on "The King's Fund," as hospital men have come to call it, King George's Fund for Sailors will, we hope, have the same expansion as its prototype. But King George's Fund was founded in an emergency when the national conscience was pricked, and one of the first duties of the new Fund is to educate the public in the habit of subscribing to marine benevolent institutions. Even now it is hard for us, for landsmen, to realise what we owe to the Navy and the mercantile marine, and to the men who man them. The debt is felt to be a peril escaped and a peril but dimly realised. But if we wish to realise the meaning of sea power (which alone saved us) let us ask a German, a neutral, even an American, and we shall be startled by their tribute to the blockade. It was a terrific weapon, the more terrible for being unseen. It exacted nerve, devotion, self-sacrifice, also unseen. Until the first list of donors to King George's Fund for Sailors becomes a growing list of annual subscribers, our country's obligation to her seamen is unfulfilled.

### THE REVIVAL OF FESTIVAL DINNERS.

As khaki disappears from the streets, and the outward signs of peace return, the hospital festival dinner is reviving. The Middlesex Hospital, which has extensive repairs on hand and is badly in need of funds, is to hold one in the autumn, at which the Prince of Wales has promised to preside. This hospital has had much experience in the successful organisation of similar social fêtes, and doubtless the forthcoming dinner will be in the tradition and offer exceptional attractions to the public. The Prince of Wales has already won a reputation in the difficult but graceful art of after-dinner speaking. No more distinguished or eloquent advocate can be found. For he will not only state his points but make them, as only an orator can.

### VALUE OF VACCINATION.

NEWS comes of an outbreak of small-pox in Croydon. It is at present fortunately of small dimensions, the cases being confined to two families. Strict precautionary measures have naturally now been taken, but it is noteworthy that, as we are informed, two of the cases occur in unvaccinated people, who now show the disease in acute form. As we write further cases have been reported in Finchley. In view of the recent discussions on the vaccination question, and the possible appearance of the disease in this country, mention of which has several times been made in this Journal, one would venture particularly to direct the attention of practitioners to this outbreak, localised though it be.

### THE MILITARY NOTE IN APPEALS.

A PERSONAL "appeal from the children" has been issued on behalf of the Royal Waterloo Hospital for Children and Women on the 103rd anniversary of the institution. It has a technical interest beside that of the work for the maintenance of which it has been issued. For it draws attention to the "casualties" on the "home front," and therefore belongs to the category of war-appeals. Such are necessarily, indeed happily, transient, and military terms will gradually disappear from institutional literature. But death and disease, though largely preventable, remain, as does the Royal Waterloo Hospital to cope with them. Yet it seems absurd that in London this hospital is in dread lest it should not succeed in raising a paltry £8,000 a year in new annual subscriptions. It cannot even convert large donations into a big annual income by purchasing an annuity, for unlike a private person it is immortal. Thus the aids and arrangements of commercial insurance are not for the Royal Waterloo Hospital, though many of its friends are people of business who benefit by the business organisation of thrift. If people would only remember its disadvantage in this respect, and consider that it is little children for whom it cares, the money should quickly be forthcoming. For the money exists ten times over, and childhood has not lost its coaxing ways.

### WAR MEMORIALS AND REPRESENTATION.

THE working classes, who are apt to mistake big committees for representative ones, seem to be trying to import "representative" questions into war memorial schemes. In no other way can we interpret the confused discussion which lately occurred over the Bishop's Stortford War Memorial. This is twofold. It embraces a scheme to improve and extend the hospital, and to erect a monument. Each scheme has its supporters. Money has been subscribed for both. But in relation to the hospital scheme the cry "more representation" has been raised. What is indeed demanded no one has yet made clear. Some say that the money should not be handed by the memorial committee to the hospital unless there is more representation. This presumably means that the memorial committee should have a representative on the hospital's board. Others complain that the hospital committee is not representative enough. To this we reply that the constitution of the hospital is not a matter which the war memorial committee was formed to discuss. Only exceptional circumstances could justify the latter in discussing it. But no exceptional circumstance, no specific grievance even, is alleged. This multiplication of committees is a mania which is really amounting to an epidemic. If the memorial committee wishes to have a say in the spending by the hospital of the memorial's funds, it is easy to arrange it. But the constitution of the hospital is a matter for its governors and subscribers to settle.

### A MEMORIAL SCHEME ABANDONED.

IT is something of a novelty to report the abandonment of a scheme to build a cottage hospital for a war memorial. The people of Hythe and Dibden in Hampshire lately decided on a cottage hospital if other parishes would join with them in raising the money for such a war memorial. Inquiries were made, but no support was found, and either Hythe and Dibden had to carry the scheme through on their own resources, or to drop it. The memorial committee reported the result of its inquiries and resigned. Perhaps the reason for the want of local support outside the two parishes is not worth much investigation. But we are sure that Hythe and Dibden will look back on their suggestion and initiative with pride in after years, and that when a new hospital is generally recognised to be necessary for the district, they will point to the clock tower which they are now about to build, and say, "We have done what we could, and our intentions were limited only by our resources."

### THE WEST LONDON WAR MEMORIAL.

WITH 160 beds for a population of threequarters of a million, the West London Hospital is hoping to extend its premises. To this end an appeal for £100,000 has been issued, some of which will have to be used to pay debts. Considerable *éclat* has attended the appeal since Mr. D. Mason, of Chiswick, a vice-president, as announced last week, has promised some £22,000 with which to provide a new out-patients' department, as a token of gratitude to the men at the Front.' Mr. G. F. Marshall, the chairman, knows that the need for more beds is not merely local; West London is a Metropolitan area, and London's need is national. Meantime the waiting list is disastrous.

### COTTAGE HOSPITALS AFTER THE WAR.

THAT cottage hospitals have suffered by the war a change "into something rich and strange," which will affect their whole future, is well seen in the case of the Southport Homœopathic Cottage Hospital; for in 1915 this was converted from a small institution for fourteen civilians into a hospital for sixty wounded. It is still wholly occupied by officer patients, and the military want its entire accommodation for some months to come. What will happen afterwards? Pre-war conditions will not entirely be restored, and in the meantime the cottage hospital not only has expanded but received such further equipment as a new electro-therapeutic installation, in connection with which the board gave to Sister Juest a course of training in London. These opportunities will hardly be wasted. In the voluntary system no true type of hospital can be spared. In- and out-patients will reap the benefit of the electrical installation, and it is hoped to devote one ward to children in co-operation with child welfare centres, and to set aside another large ward for soldiers and sailors suffering from the after-effects of the war. A fixed scale of payments is to be arranged. The only point of the programme which suggests misgiving is that subscribers' "recommends" will have definite value. If this means that the bad old ticket or letter system is to be extended, it is open to criticism. That system, and the voting which in large institutions depends upon it, is, as we have often said, petty patronage in its last ditch, and causes delay, suffering, and disappointment out of all proportion to its supposed financial advantage.

### A FORTUNATE CONVALESCENT HOME.

On the closing of the Gledhow Hall V.A.D. Hospital, Yorkshire, a sum of approximately £3,000 was in hand, and this has been handed to the committee of the Meanwood Convalescent Home for Children, Leeds. The gift comes at the right time, for it will enable certain important alterations to be made. Separate accommodation for children with infectious diseases is perhaps the chief. What the Home needs is extension and modernisation; and already the Medical Officer of Health has urged the committee to keep the Home open all the year round. The memory of the V.A.D. Hospital which has provided these funds will not be lost. Miss Cliff, the commandant, in making the gift said that the nurses, the staff, and the soldier patients agree in wishing that the Home should bear the hospital in mind, and that the soldiers' children should have some special benefit. A stone inscription has been suggested to commemorate the 2,500 men who have passed through the hospital's wards.

### HOSPITALS AND THE SPIRIT DUTY.

Hospital pharmacists are keenly awaiting the decision of the Government regarding the largely increased duty on alcohol imposed by the Budget, 1919, and its application to spirituous preparations, such as tinctures, etc., for medical use. In a previous Budget, owing mainly to the efforts of Sir W. Glyn-Jones, Secretary and Registrar of the Pharmaceutical Society, an abatement in the duty of 15s. 9d. per proof gallon was made where alcohol was used for making strictly medical preparations. Hospitals were thus exempted from a highly increased expenditure on medicines, though in practice the duty had to be paid and abatement claimed afterwards. We understand that the matter is now engaging the attention of the Pharmaceutical Society, and it is hoped that their representatives will convince the Chancellor of the Exchequer that there is a good case for continuing the policy of making an abatement on alcohol used for medicinal preparations by hospitals and druggists.

### THE STAFF-SURGEON: A DEPARTURE.

An important proposal is being considered by the governors of the West Norfolk and King's Lynn Hospital, namely that, while retaining the honorary services of the physicians, a surgeon should be appointed at a salary sufficient to enable him to undertake all the surgical work of the hospital, and so to free him from private practice, except as an operator. An F.R.C.S. is available, who is willing to abandon general practice and to devote his whole time to surgery; one who is not imported from without, but who would merely be giving to the hospital more exclusive services than hitherto. The arrangement seeks to improve the work of the hospital, to give a more formal standard to its surgery, and to find a mean, for the present, between an entirely honorary and a fully-paid staff. It may suggest to other hospitals of similar size and like circumstances a change which all so placed and equally ambitious will do well to consider.

### INCURABLE COLCHESTER.

Colchester, if we remember, has often been reminded by the authorities of the Essex County Hospital of its want of public spirit in support of that institution. Certain classes have done their share, but the mass of the inhabitants never. Persons of note have echoed the committee's warning. It has been disregarded as before. Now the people of the town and county are confronted with a very big bill, the accumulated cost of years of neglect. First comes Mr. Paul Waterhouse, the distinguished architect, who knows the meaning of economy as only a true architect can. After due consideration, he puts the cost of reconstructing the hospital at £50,000. There can be no charge of extravagance there. Next comes the cessation of the revenue from military patients, a serious annual loss. Then follows the Treasurer's estimate of an annual deficit of £3,000 a year. In the distance is the figure of the Minister of Health, who is certain to throw more work on the hospital than it is now doing, or has done, because of the inertia of many Colchester people. The consequence is that they will have to foot a very big bill, and raise further money for maintenance when the construction, which cannot be long delayed, inevitably takes place. It is a large order, and the Colchester folk as a community richly deserve the burden they have brought upon themselves.

### BOLINGBROKE HOSPITAL £10,000 FUND.

Some months ago the Mayors of Battersea and Wandsworth made a joint appeal for £10,000 towards the funds of the Bolingbroke Hospital. Though the £10,000 has not been raised, the united efforts have met with some success. Already £2,000 out of the £10,000 desired has been handed over to the hospital authorities, and, as the result of collections on St. George's Day, nearly £700 is ready towards the third £1,000. It is hoped that by the end of the year £5,000, or one half of the amount the Mayors set out to raise, will have been handed over. Many firms have decided to ask their employees to give a small weekly sum to the fund. The general adoption of this plan would greatly benefit the fund.

### THE SOUTH LONDON HOSPITAL.

Steady work is bringing its reward to the South London Hospital for Women, which has received a gift of £4,000 from Lord Cowdray. This should help in the extension which is needed, for the money is to be devoted to the perpetual endowment of two single-bed wards. One will be called the Cowdray ward, and the second the Paddock Hurst ward. Already the hospital's statistics imply a considerable range of work. The number of out-patient attendances last year run well into five figures. and there were 1,173 in-patients. We believe that the scheme was launched just in time to benefit by the proposed Ministry of Health and not too late to be swamped by its coming organisation.

### A NEW TRAINING HOSPITAL

On June 1 the "John Leigh" Memorial Hospital, Altrincham, will be opened under the Ministry of Pensions as a treatment and training hospital for officers. Disabled officers will be accommodated in one half. The other will be occupied by officers who, though under treatment, are able to attend vocational schemes in Manchester and the neighbourhood. The new hospital, which is available through the generosity of Sir John Leigh, Bart., has been used by the Red Cross for 100 officers during the war.

### ST. GEORGE'S HOSPITAL.

The latest endeavour to solve the problem of the future of St. George's Hospital is the decision to rebuild on the present site, though, as everyone knows, the opportunities there are limited. Since the withdrawal just before the War of the offer of £460,000 for the present site of St. George's the matter has not advanced, and neither the amalgamation with Westminster Hospital, nor the movement of the twain to Wandsworth Bridge has come to anything. The present decision looks like a decision *faute de mieux*, and can hardly claim the applause of statesmanship. But the future of Westminster Hospital? That cannot hang much longer in expectancy.

### PREVENTIVE MEDICINE AT CARDIFF.

Thanks to the liberality and foresight of the late Miss Talbot, who used her great wealth so very wisely, the young medical school at Cardiff is now in a position to light a path in which more venerable foundations will need very shortly to follow, if they are not to be outdone by their junior rival. In a word, Lord Aberdare, Sir William Osler, and Sir Arthur May have nominated to the Professorship of Preventive Medicine at Cardiff College Dr. E. L. Collis, formerly of the Home Office inspectorate, and more recently Director of Welfare and Health in the Ministry of Munitions. Valuable as is the work which Dr. Collis has already done in preventive medicine, it is natural to expect that his new opportunities will be greater than anything he has enjoyed before, and that they will be duly fruitful of results. The details of the scheme drawn up by the trustees are not to hand at the moment; but the stipend of the chair is stated to be £1,500 a year—not a penny too much, though more than most professors get in this country. Still, a beginning has been made which may help in time to remove one of the standing reproaches against British medical education.

### AN ECONOMICAL MATRON

It is not often, we suppose, that the President of a Cottage Hospital is Lord Mayor of London, but that is one fact of which Purley Cottage Hospital may this year be proud. Another concerns its first matron, Miss Beard, who has resigned from ill-health. A remarkable tribute was paid her by Mr. D. M. Kerly in a recent speech. She was not only capable and devoted, he said, but a cheery presence in the hospital, and the value of her work as an economist could be judged from the fact that when she was away for three months the expenses nearly doubled, so it was obvious that the committee was not anxious to part with her. All regretted that the strain of the work had been too much, but glad to know that her rest had been beneficial.

### CHILD WELFARE IN HERTFORDSHIRE.

In connection with the maternity and child welfare scheme of the Hertfordshire County Council, the Health Committee has prepared a scheme which involves the purchase of the Hertfordshire Children's Convalescent Home at St. Leonards-on-Sea for the sum of £2,000, to provide accommodation for certain cases after confinement, and for the after-care of children suffering from measles, whooping cough, and other illnesses.

### THIS WEEK'S DRUG MARKET.

The improved tone in the drug trade is maintained, and there are indications that business is gradually getting into something like normal shape. Buying on a fair scale is going on, and the consequence is that a brake has been put on the downward tendency of prices. As a matter of fact, the list of drugs with an upward price tendency is this week larger than the list of those whose values have a contrary direction: camphor, menthol, peppermint oil, cloves, clove oil, ergot of rye, castor oil, saccharin, resorcin, balsam tolu, citric acid, linseed oil, and phenacetin are the principal articles whose price tendency is upwards. On the other hand, salicylic acid, aspirin, creosote, and potassium permanganate still tend downwards. Prices are a long way from being stable, and caution in making purchases is advised.

